# O Treinamento Físico Resistido Atenua as Disfunções Ventriculares Esquerdas em Modelo de Hipertensão Arterial Pulmonar

**DOI:** 10.36660/abc.20210681

**Published:** 2022-08-24

**Authors:** Leôncio Lopes Soares, Luciano Bernardes Leite, Luiz Otávio Guimarães Ervilha, Bruna Aparecida Fonseca da Silva, Maíra Oliveira de Freitas, Alexandre Martins Oliveira Portes, Leonardo Mateus Teixeira Rezende, Filipe Rios Drummond, Miguel Araújo Carneiro, Mariana Machado Neves, Emily Correna Carlo Reis, Antônio José Natali

**Affiliations:** 1 Universidade Federal de Viçosa Departamento de Educação Física Viçosa MG Brasil Universidade Federal de Viçosa , Departamento de Educação Física , Viçosa , MG – Brasil; 2 Universidade Federal de Viçosa Departamento de Biologia Geral Viçosa MG Brasil Universidade Federal de Viçosa , Departamento de Biologia Geral , Viçosa , MG – Brasil; 3 Universidade Federal de Viçosa Departamento de Medicina Veterinária Viçosa MG Brasil Universidade Federal de Viçosa , Departamento de Medicina Veterinária , Viçosa , MG – Brasil

**Keywords:** Insuficiência Cardíaca, Hipertensão Pulmonar, Ratos, Condicionamento Físico Animal/métodos, Miócitos Cardíacos, Disfunção Ventricular Esquerda, Exercício

## Abstract

**Fundamento:**

A hipertrofia e a dilatação do ventrículo direito observadas na hipertensão arterial pulmonar (HAP) prejudicam a dinâmica do ventrículo esquerdo (VE) achatando o septo interventricular.

**Objetivo:**

Investigar se o treinamento físico resistido (TFR) de intensidade baixa a moderada é benéfico para funções contráteis do VE e de cardiomiócitos em ratos durante o desenvolvimento de HAP induzida por monocrotalina (MCT).

**Métodos:**

Foram usados ratos Wistar machos (Peso corporal: ~ 200 g). Para avaliar o tempo até o possível surgimento de insuficiência cardíaca (ou seja, ponto de desfecho), os ratos foram divididos em dois grupos, hipertensão com sedentarismo até a insuficiência (HSI, n=6) e hipertensão com treinamento até a insuficiência (HTI, n=6). Para testar os efeitos do TFR, os ratos foram divididos entre grupos de controle sedentários (CS, n=7), hipertensão com sedentarismo (HS, n=7) e hipertensão com treinamento (HT, n=7). A HAP foi induzida por duas injeções de MCT (20 mg/kg, com um intervalo de 7 dias). Os grupos com treinamento foram submetidos a um protocolo de TFR (subir escadas; 55-65% da máxima carga carregada), 5 dias por semana. A significância estatística foi definida em p <0,05.

**Resultados:**

O TFR prolongou o ponto de desfecho (~25%), melhorou a tolerância ao esforço físico (~55%) e atenuou as disfunções de contratilidade de VE e de cardiomiócitos promovidas pela MCT preservando a fração de ejeção e o encurtamento fracional, a amplitude do encurtamento, e as velocidades de contração e relaxamento nos cardiomiócitos. O TFR também preveniu os aumentos de fibrose e colágeno tipo I no ventrículo esquerdo causados pela MCT, além de manter as dimensões de miócitos e colágeno tipo III reduzidas por MCT.

**Conclusão:**

O TFR de intensidade baixa a moderada é benéfico para funções contráteis de VE e cardiomiócitos em ratos durante o desenvolvimento de HAP induzida por MCT.

## Introdução

Aumentos na resistência da vasculatura pulmonar, causados principalmente pela disfunção endotelial, levam à hipertensão arterial pulmonar (HAP). ^1^ A resistência da vasculatura pulmonar sobrecarrega o ventrículo direito resultando em remodelação patológica, ^2^ e disfunção devido a hipertrofia e dilatação. ^1^ Essa remodelação afeta a dinâmica do ventrículo esquerdo (VE) por causa da interação ventricular direta. Nessa estrutura, a dinâmica do ventrículo esquerdo é prejudicada pelo achatamento do septo interventricular, ^3 , 4^ pois ela enfrenta enchimento diastólico precoce deficiente, volume diastólico final reduzido e remodelação adversa. ^3 , 5 , 6^ Portanto, pacientes com HAP apresentaram um volume de acidente vascular cerebral ^3^ e tolerância a esforço físico reduzidos, o que afeta negativamente sua qualidade de vida e sobrevida. ^7^

Os tratamentos farmacológicos têm o objetivo de reduzir a pressão arterial pulmonar e a sobrecarga no ventrículo direito, e, assim, manter a função cardíaca. ^8^ Já se demonstrou que pacientes com HAP podem manter a função cardíaca por meios não farmacológicos, tais como a prática regular de exercícios físicos. ^9 , 10^ No modelo experimental de HAP grave induzida por monocrotalina (MCT), por exemplo, o exercício aeróbico prévio ou precoce demonstrou promover benefícios cardiovasculares, tais como a atenuação da hipertrofia, a disfunção e a remodelação adversa do ventrículo direito. ^11 - 16^ Nosso grupo de pesquisa ^17 , 18^ relatou recentemente que a corrida voluntária (ou seja, treinamento de alta intensidade intermitente) adia o início da insuficiência cardíaca, e alivia a remodelação adversa do ventrículo direito e a disfunção de miócitos (ou seja, a contratilidade de miócitos e a deterioração da ciclagem de Ca ^2^ intracelular) nesse modelo. Além disso, também demonstramos que o exercício aeróbico contínuo de intensidade moderada previne a remodelação adversa do ventrículo direito, a contratilidade de miócitos e as deficiências da ciclagem de Ca ^2^ . ^19^

O uso de treinamento físico resistido (TFR) de intensidade baixa a moderada foi recomendado como parte de programas de treinamentos para promover a saúde e prevenir doenças cardiovasculares ^20 , 21^ incluindo as relacionadas à disfunção do ventrículo esquerdo. ^22^ Em relação à HAP, intervenções combinadas de exercícios, incluindo aeróbicos, treinamento de resistência e treinamento muscular inspiratório específico, demonstraram ser seguras para esses pacientes e resultaram em melhorias significativas na potência muscular, na capacidade de exercício e na sobrevida. ^23 - 25^ Apesar disso, embora o exercício aeróbico tenha demonstrado evitar a disfunção diastólica e sistólica do ventrículo esquerdo nas condições de linha de base e isovolumétrica, ^26^ na HAP induzida por MCT, o impacto do TFR na disfunção do ventrículo esquerdo nesse modelo é desconhecido.

Embora os modelos animais tenham embasado a descoberta de novas terapias e o entendimento da fisiopatologia da HAP, o modelo de lesão pulmonar do MCT em roedores usando a injeção de 60 mg/kg de massa corporal induz HAP grave em um processo subagudo, que é limitado para simular a HAP crônica humana. ^27^ Nesse sentido, Whang et al., ^28^ demonstraram que 40 mg/kg MCT divididos em duas injeções de 20 mg/kg com um intervalo de sete dias são o que melhor imitam HAP crônica com as alterações comuns na estrutura e função das artérias pulmonares e do ventrículo direito observadas em seres humanos. Portanto, no presente estudo, usamos esse modelo em ratos para testar se o TFR de intensidade baixa a moderada é benéfico para o VE e para funções contráteis em miócitos durante o desenvolvimento de HAP induzida por MCT. A hipótese deste estudo é de que o TFR de intensidade baixa a moderada é benéfico para funções contráteis de VE e miócitos em ratos durante o desenvolvimento de HAP induzida por MCT.

## Métodos

### Desenho do experimento e indução da HAP

Após a definição do tamanho da amostra, ^29^ trinta e três ratos Wistar machos [Peso corporal: ~200 g] foram obtidos do laboratório de animais da Universidade Federal de Viçosa, MG, Brasil. Os animais foram mantidos em gaiolas de policarbonato transparentes, em uma sala com controle de temperatura (~22 °C) e ~60% de umidade relativa, em ciclos de 12/12 h de luz/escuro, e tiveram acesso livre a água e ração comercial.

Para avaliar o tempo até o possível surgimento de insuficiência cardíaca, 12 animais (~200 g) foram divididos em dois grupos, usando a randomização simples, hipertensão com sedentarismo até a insuficiência (HSI, n=6) e hipertensão com treinamento até a insuficiência (HTI, n=6). Após as injeções de MCT, ratos dos grupos HSI e HTI foram eutanasiados quando apresentaram sinais clínicos externos previamente validados de possível surgimento de insuficiência cardíaca (por exemplo, perda de peso, dispneia, piloereção) e não podiam mais se alimentar adequadamente, subir a escada (grupo HTI) ou mesmo se mover dentro da gaiola, ^30 - 37^ que foi considerado um ponto de desfecho.

Para testar se os efeitos do TFR eram benéficos durante o desenvolvimento da HAP, 21 animais (~200 g) foram divididos entre grupos usando a randomização bloqueada: de controle sedentários (CS, n=7), hipertensão com sedentarismo (HS, n=7) e hipertensão com treinamento (HT, n=7). Animais dos grupos HS, HT e CS foram eutanasiados no dia do ponto de desfecho mediano (± 1 dia) dos animais do grupo HSI (ou seja, 28 dias). O tempo mediano até o possível surgimento de insuficiência cardíaca representou o momento após o tratamento com MCT em que mais de 50% do grupo chegou ao dia do ponto de desfecho. Os animais nos grupos com treinamento foram submetidos a TFR, e aqueles nos grupos sedentários foram mantidos em suas gaiolas.

Para induzir a HAP, os animais dos grupos HSI, HTI, HS e HT receberam 2 injeções intraperitoneais de MCT (Sigma-Aldrich, USA) de 20 mg/kg, com intervalos de 7 dias para induzir a insuficiência do ventrículo direito. ^28^ Os animais de controle receberam injeções de solução salina em volume equivalente.

Os experimentos foram conduzidos de acordo com procedimentos internacionais para pesquisa com animais (Scientific Procedures; Act 1986). Todos os protocolos foram analisados e aprovados pelo Comitê de Ética Institucional (número de protocolo 02/2019).

### Treino de resistência e teste de carga máxima

Os animais foram acostumados com o protocolo de TFR (adaptado de Hornberger e Farrar) ^38^ por uma semana antes da primeira injeção de MCT ou solução salina, sem carga adicional. O TFR consistiu em subir uma escada (1,1 m de altura, inclinação de 80º) com intervalos de repouso de 2 minutos, sendo que a carga foi baseada em um teste de máxima carga carregada. O teste de máxima carga carregada foi realizado antes da injeção de MCT ou solução salina (tempo 0) e no 14º, 21º e 28º dia após as injeções. O teste consistiu em subir escada com carga inicial de 75% do peso corporal, que foi gradualmente aumentado e mais 15% nas subidas subsequentes até que o animal não pudesse mais subir. ^39^ A carga foi presa à cauda do rato e as subidas foram intercaladas com 2 minutos de intervalos de repouso. A máxima carga carregada foi usada como índice de tolerância de esforço físico.

Os animais exercitados foram submetidos a um programa de TFR, 5 vezes por semana durante o período experimental até o dia anterior ao da eutanásia, totalizando vinte sessões de exercício. A carga do TFR foi de 55-65% da máxima carga carregada seguindo as recomendações para pacientes com doenças cardiovasculares. ^20^ Cada sessão de treinamento consistiu em 15 subidas intercaladas com intervalos de 60 segundos, sendo que a carga de treinamento foi ajustada após os testes de máxima carga carregada (14º e 21º dia).

### Ecocardiografia e coleta de amostras

As avaliações ecocardiográficas foram realizadas no 28º dia após a primeira injeção com MCT. Os animais foram anestesiados (Isoflurane 1,5% e100% de oxigênio em um fluxo constante de 1L/min; Isoflurane, BioChimico, Brasil) e as imagens foram obtidas enquanto os animais estavam em decúbito lateral. Estudos bidimensionais com uma taxa de amostragem rápida de 120 fps no modo M foram realizados usando o sistema de ultrassom MyLabTM30 (Esaote, Gênova, Itália) e transdutores de frequência nominal de 11 MHz. A ecocardiografia transtorácica bidimensional e modo M foram obtidos numa velocidade de digitalização de 200 mm ajustado de acordo com a frequência cardíaca. ^40^ Para avaliar a função do VE, os seguintes parâmetros foram avaliados: fração de ejeção (FE) do VE; e encurtamento fracional (EF). Para caracterizar a HAP, a excursão sistólica do plano anular tricúspide (TAPSE) foi determinada.

No dia do desfecho final mediano (± 1 dia) dos animais do grupo HSI, animais dos grupos HS, HT e CS foram eutanasiados. Após a eutanásia, animais dos grupos CS, HS e HT tiveram seus corações, ventrículos e pulmões dissecados, pesados e processados para análises de interesse, conforme descrito abaixo. A tíbia direita foi dissecada, e seu comprimento foi medido.

### Histomorfometria

As análises histológicas do VE foram realizadas conforme descrito anteriormente. ^41 , 42^ Rapidamente, imediatamente após a coleta, fragmentos do VE foram fixados com o fixador Karnovsky (paraformaldeído a 4% e glutaraldeído a 4% em tampão de fosfato a 0,1 M, pH 7,4) por 24 horas. Em seguida, os fragmentos foram desidratados em etanol, clarificados em xilol e embebidos em parafina. Os blocos foram cortados em seções de 5 μm de espessura, montados em lâminas histológicas e corados com hematoxilina e eosina para medir a área da seção transversal (AST), ou com vermelho Sirius para contar fibras de colágeno e/ou com tricromo de Masson para contagem de fibrose cardíaca. Para evitar as análises repetidas da mesma área histológica, as seções foram avaliadas em semisséries, usando uma em cada 10 seções. As imagens digitais das lâminas coradas com vermelho Sirius foram obtidas usando um microscópio de luz polarizada (Olympus AX-70, Tóquio, Japão) conectado a uma câmera digital (Olympus Q Color-3, Tóquio, Japão) e as imagens das lâminas coradas com hematoxilina e eosina e tricromo de Masson foram obtidas usando um microscópio de luz (Olympus AX-70, Tóquio, Japão) conectado a uma câmera digital (Olympus Q Color-3, Tóquio, Japão). A quantificação dos tipos de colágeno e fibrose cardíaca foi realizada usando-se uma ferramenta de identificação de cores específica usando o software Image-Pro Plus 4.5 (Media Cybernetics, Silver Spring, MD, EUA). A área de seção transversal do miócito foi medida usando-se uma ferramenta específica (medição manual em software Image Pro-Plus 4.5).

### Isolamento de miócitos no ventrículo esquerdo

O coração foi conectado a um sistema de perfusão retrógrada de Langendorff e miócitos simples do VE isolados conforme descrito anteriormente. ^18^ Resumidamente, o sistema de perfusão do coração acontece via aorta com solução de Tyrode contendo (em mM; Sigma-Aldrich, EUA): 130 NaCl, 1,43 MgCl2, 5,4 KCl, 0,75 CaCl2, 5,0 Hepes, 10,0 glicose, 20,0 taurina e 10,0 creatina, pH 7,4 por cerca de 5 minutos. A solução de Tyrode foi substituída por uma solução de Tyrode contendo EGTA (0,1 mM) por 6 minutos. Em seguida, foi feita a perfusão do coração com solução de Tyrode contendo 1 mg/ml de colagenase tipo II (Worthington, EUA) e 0,1 mg/ml de protease (Sigma-Aldrich, EUA) por cerca de 12 minutos. Em seguida, o VE do coração digerido foi retirado e cortado em pequenos fragmentos que foram colocados em um frasco cônico contendo a solução enzimática (colagenase e protease). As células foram mecanicamente separadas agitando-se o frasco por 5 minutos. As células dispersadas foram separadas do tecido não dispersado por filtragem por centrifugação. As células isoladas foram armazenadas a 5° C até o uso. Os miócitos isolados foram usados de 2 a 3 horas após o isolamento. As soluções usadas no procedimento de isolamento foram oxigenadas (O _2_ 100% – White Martins, Brasil) e mantidas a 37° C.

### Função contrátil de miócito simples

A função contrátil de miócitos do VE foi medida usando-se um sistema de detecção de ponta (Ionoptix, Milton, EUA) montado em um microscópio invertido (Nikon Eclipse – TS100, Japão) conforme descrito anteriormente. ^19^ Os miócitos foram colocados em um banho na mesa de um microscópio invertido e superfusados com uma solução de Tyrode contendo, em mM (Sigma-Aldrich, EUA): 137 NaCl, 5,4 KCl, 0,33 NaH _2_ PO _4_ , 0,5 MgCl _2_ , 5 HEPES, 5,6 glicose 1,8 CaCl _2,_ pH 7,4 com 5N NaOH, a 37˚ C. Apenas os miócitos que apresentam um padrão de estriação claro e regular (sarcômero), sem contração espontânea na ausência de estimulação externa e respondendo a estimulação de 1Hz com uma única contração foram testados. Os miócitos foram estimulados (Myopacer, Ionoptix, Milton, EUA) para se contrair a uma frequência de estimulação progressiva (1, 3, 5 e 7 Hz) usando eletrodos externos, e o encurtamento de célula resultante medido por análise de uma imagem de vídeo da célula usando-se a câmera e o software Ionoptix (Ionoptix, Milton, MA, EUA). O encurtamento da célula foi expresso como % do comprimento da célula em repouso.

O comprimento e a largura do miócito foram obtidos a partir da imagem de vídeo da célula; e o volume da célula foi calculado conforme descrito anteriormente. ^43^

### Análise estatística

A normalidade dos dados foi testada usando-se o teste de Shapiro-Wilk. Os dados são apresentados como médias ± DP, para variáveis contínuas com distribuição normal, como mediana e faixa interquartil, para variáveis contínuas sem distribuição normal. O ponto de desfecho, os parâmetros de remodelação ventricular e os parâmetros contráteis de cardiomiócitos isolados apresentaram uma distribuição não normal, enquanto os parâmetros de exercício, peso corporal e dos órgãos e área da seção transversal e morfometria da célula isolada apresentaram distribuição normal. O ponto de desfecho foi testado pela curva de análise de Kaplan-Meier pelo teste de Log-rank. A máxima carga carregada, o peso corporal, a função do VE, o peso dos órgãos e os parâmetros de célula simples foram testados pela análise de variância de via única (ANOVA) ou de Kruskal-Wallis seguida de teste post-hoc de Dunn. A máxima carga carregada foi testada por medidas de ANOVA de via única repetidas. As análises ANOVA foram seguidas do teste de correção de Tukey pareado. O teste Qui-quadrado de Pearson (X ^2^ ) foi usado para avaliar a proporção de animais que apresentaram achatamento do septo interventricular. A significância estatística foi definida em P <0,05. A descrição dos dados, o número de ratos e miócitos são apresentados nas legendas das tabelas e das figuras. Todas as análises foram realizadas utilizando o software GraphPad Prism, versão 6.01 (San Diego, CA, EUA).

## Resultados

### Possível surgimento de insuficiência cardíaca e tolerância ao esforço físico

**Figura 1:**
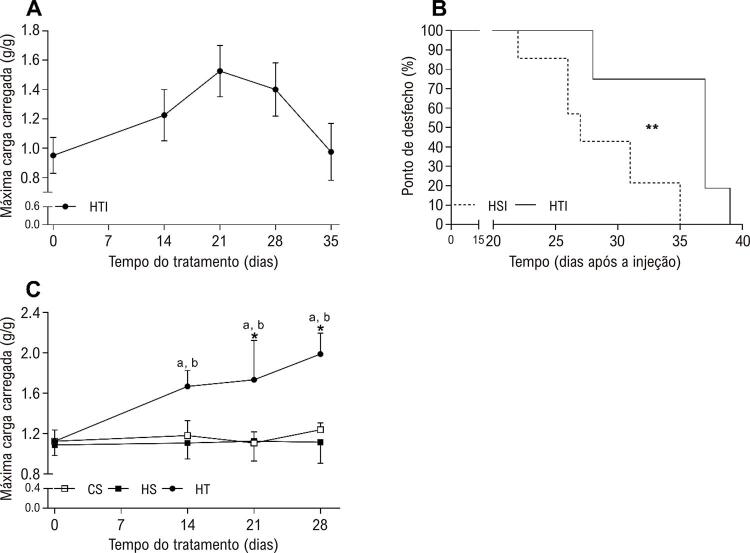
Efeito do treinamento resistido em possível surgimento de insuficiência cardíaca (ponto de desfecho) e tolerância ao esforço físico. (A) Máxima carga carregada de animais hipertensos até a insuficiência, determinada pela máxima carga carregada ajustada para peso corporal, antes da injeção (dia 0) e no 14º, 21º e 28º e 35º dias após a primeira injeção de monocrotalina. (B) Ponto de desfecho, medido em dias para apresentar sinais de possível surgimento de insuficiência cardíaca, foi significativamente mais curto em ratos em hipertensão com sedentarismo até a insuficiência (HSI, n=6) do que nos ratos em hipertensão com treinamento até a insuficiência (HTI, n=6). **P <0,01, análise da curva de Kaplan-Meier pelo teste de Log-rank. (C) Máxima carga carregada relativa para animais de controle, hipertensos, sedentários e em treinamento, determinada conforme o painel A. Ratos em hipertensão com treinamento (HT, n=7) apresentaram maior ganho em carga carregada do que o grupo de controle sedentário (CS, n=7) e o grupo de hipertensão com sedentarismo (HS, n=7), a partir do 14º dia. Medições de ANOVA repetidas seguidas do teste de correção de Tukey. aP <0,05 vs. HS; bP <0,05, vs. CS; *P <0,05 vs. antes da injeção de MCT.

A figura 1A ilustra que os animais do grupo HTI realizaram o protocolo de treinamento de resistência durante o desenvolvimento da HAP até apresentar sinais do possível surgimento de insuficiência cardíaca. A máxima carga carregada aumentou progressivamente até o 21º dia e, depois disso, diminuiu até o nível inicial no 35º dia após a primeira injeção de MCT. Todos os animais dos grupos HSI e HTI apresentaram sinais de possível surgimento de insuficiência cardíaca - ponto de desfecho ( Figura 1B ); entretanto, os animais do grupo HTI tiveram um tempo mediano de ponto de desfecho mais longo (37 dias) do que aqueles no grupo de HSI (28 dias), indicando benefícios do treinamento resistivo.

Ratos hipertensos do grupo de HT melhoraram sua tolerância ao esforço físico ( Figura 1C ) durante o experimento. A máxima carga carregada no grupo HT foi mais alta no 21º e no 28º dias do que no dia 0. Além disso, esses animais tiveram a máxima carga carregada mais alta no 14º, 21º e 28º, comparados aos dos grupos CS e HS.

### Função e morfologia do ventrículo esquerdo

**Figura 2:**
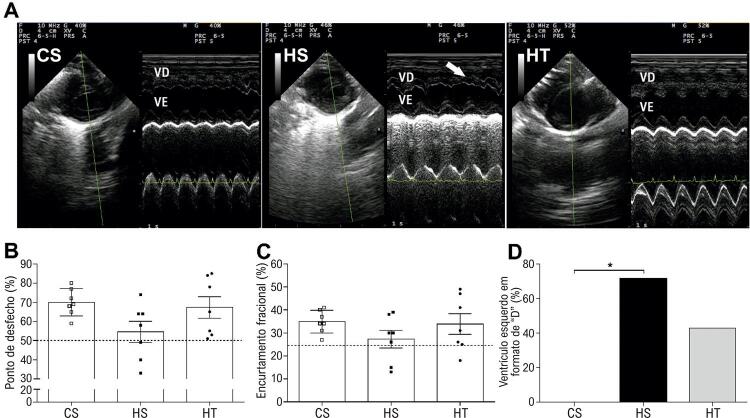
Efeito do treinamento físico resistido na função do ventrículo esquerdo avaliado no 28º dia após a primeira injeção de monocrotalina. (A) Imagens ecocardiográficas representativas (B) Fração de ejeção (C) Encurtamento fracional. (D) Ventrículo esquerdo em forma de “D”. Os valores são médias ± DP (n=7 ratos em cada grupo), CS: controle sedentários; HS: hipertensão com sedentarismo; HT: hipertensão com treinamento; VD: ventrículo direito; VE: ventrículo esquerdo. A linha pontilhada indica os limites para a classificação da função prejudicada. Painéis B e C: ANOVA de uma via seguido de teste post hoc de Tukey. Painel D: Teste Qui-quadrado de Pearson (teste x2). *p <0,05.

A avaliação ecocardiográfica mostrou o achatamento do septo interventricular chamado de ventrículo esquerdo em forma de “D” em animais dos grupos HS e HT ( Figura 2A ), o que sugere sobrecarga de pressão do ventrículo direito, característica da HAP. Essa alteração morfológica foi maior no grupo HS do que no grupo CS, enquanto o grupo HT apresentou valores intermediários ( Figura 2D ). Em relação à função do ventrículo esquerdo, não se encontrou nenhuma diferença entre os grupos para a fração de ejeção ( Figura 2B ) ou ao encurtamento fracional ( Figura 2C ). Apesar disso, é importante observar que 3 dos 7 animais do grupo HS tiveram fração de ejeção <50% e 3 de 7 tiveram encurtamento fracional <25%, indicativo de insuficiência do ventrículo esquerdo. Por outro lado, nenhum dos animais treinados (HT), no mesmo período, apresentaram fração de ejeção <50% e apenas 1 de 7 teve encurtamento fracional <25%.

A presença de HAP em animais do grupo HS foi caracterizada também pelos valores de TAPSE. Animais do grupo HS apresentaram valores de TAPSE mais baixos (1,43 ± 0,23) do que os dos grupos CS (2,06 ± 0,17) e HT (2,13 ± 0,36).

Os animais do grupo HS apresentaram menor peso corporal do que os dos grupos CS e HT ( Tabela 1 ). Apesar de não haver diferença de peso do coração entre os grupos, os animais dos grupos HS e HT tiveram maior peso do ventrículo direito (VD) e proporção entre ventrículo direito e tíbia do que os animais do grupo CS, o que indica a hipertrofia do ventrículo direito. Embora o peso do pulmão e a proporção entre pulmão e tíbia serem maiores nos grupos HS e HT do que no grupo CS, não foi encontrada diferença entre peso de VE e proporção peso de VE e tíbia entre os grupos. Em relação às dimensões dos miócitos do ventrículo esquerdo, o grupo HS apresentou comprimento, largura e volume menores do que o grupo CS. O grupo HT apresentou valores intermediários entre os dos grupos CS e HS. Os animais dos grupos de HS apresentaram AST mais baixa em comparação com os animais dos grupos CS e HT. Por outro lado, não houve diferença na AST dos animais do grupo HT em comparação com os animais do grupo CS, sugerindo um efeito benéfico do programa de treinamento resistido para prevenir a remodelação adversa do ventrículo esquerdo.

**Tabela 1:** Efeito do treinamento físico resistido no corpo e no peso dos órgãos

	CS	HS	HT
Peso corporal final (g)	298,6 ± 19,01	276,3 ± 19,87 ^*^	303,7 ± 20,98 ^†^
Peso do coração (g)	1,23 ± 0,11	1,30 ± 0,18	1,28 ± 0,12
Peso do VD (g)	0,33 ± 0,04	0,42 ± 0,03 ^*^	0,44 ± 0,05 ^*^
Peso do VE (g)	0,74 ± 0,10	0,65 ± 0,07	0,70 ± 0,07
Peso do pulmão (g)	1,65 ± 0,28	2,77 ± 0,41**	2,38 ± 0,33**
Razão entre peso do VD e comprimento da tíbia (g/cm)	0,09 ± 0,01	0,11 ± 0,00*	0,11 ± 0,01*
Razão entre peso do VE e comprimento da tíbia (g/cm)	0,20 ± 0,02	0,17 ± 0,02	0,18 ± 0,02
Razão entre peso do pulmão e comprimento da tíbia (g/cm)	0,45 ± 0,10	0,73 ± 0,11**	0,63 ± 0,09*
Comprimento do miócito (µm)	132,3 ± 19,09	122,5 ± 19,86**	129,2 ± 21,42
Largura do miócito (µm)	46,12 ± 10,08	41,75 ± 9,95*	43,64 ± 9,50
Volume do miócito (pL)	46,24 ± 3,97	38,71 ± 3,18**	42,62 ± 3,61
Seção transversal do miócito (µm ^2^ )	462,1 ± 21,86	400,5 ± 43,34*	492,2 ± 66,56 ^†^

Os valores são médias ± DP de 7 ratos e 10 células em cada grupo. CS: controle sedentários; HS: hipertensão com sedentarismo; HT: hipertensão com treinamento; VD: ventrículo direito; VE: ventrículo esquerdo; ^*^ p <0,05 vs. CS; ^**^ p <0,01 vs. CS; ^†^ p <0,05 vs. HS. ANOVA de uma via seguido de teste post hoc de Tukey.

### Remodelação adversa do ventrículo esquerdo

**Figura 3:**
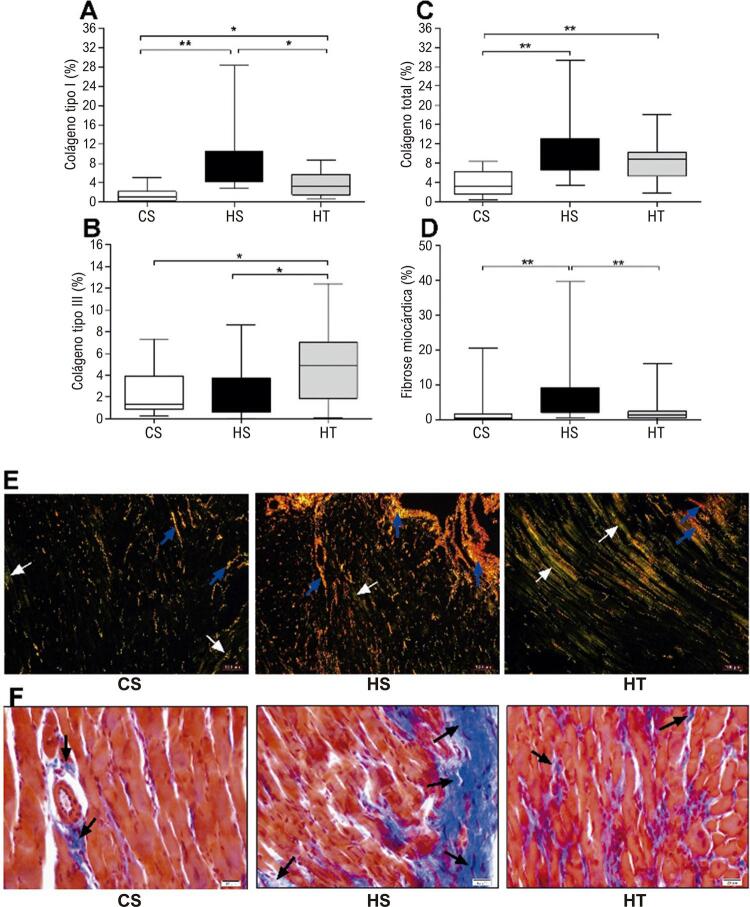
Efeito do treinamento físico resistido na remodelação do ventrículo esquerdo. (A) Porcentagem de colágeno tipo I. (B) Porcentagem de colágeno tipo III. (C) Porcentagem de colágeno total. (D) Porcentagem de fibrose no VE. (E) Microfotografias representativas do tecido do VE corado com vermelho Sirius; (F) Microfotografias representativas do tecido do VE corado com tricromo de Masson. A seta azul indica colágeno tipo I; A seta branca indica colágeno tipo III; A seta preta indica fibrose cardíaca. Os valores são apresentados como medianas acompanhadas da faixa interquartil de 10 imagens por animal em cada grupo (n=5 ratos em cada grupo). CS: controle sedentários; HS: hipertensão com sedentarismo; HT: hipertensão com treinamento. Kruskal-Wallis, seguido pelo teste post-hoc de Dunn: * p <0,05, e ** p <0,01.

A figura 3 mostra dados sobre as fibras de colágeno e a fibrose do VE. Animais hipertensos (HS e HT) apresentaram uma alta porcentagem de colágeno tipo I em comparação aos animais do grupo de controle (CS) ( Figura 3A ). Entretanto, os animais do grupo HT tiveram uma porcentagem menor de colágeno tipo I em comparação com os animais do grupo HS, mostrando o efeito protetor do TFR no avanço da HAP. Além disso, os animais do grupo TH apresentaram uma porcentagem mais alta de colágeno tipo III do que a dos animais sedentários (CS e HS) ( Figura 3B ). Animais hipertensos (HS e HT) tiveram uma alta porcentagem de colágeno total em comparação aos animais do grupo de controle ( Figura 3C ). Em relação à fibrose do VE ( Figura 3D ), os animais do grupo HS apresentaram uma porcentagem mais alta, em comparação com os animais nos grupos CS e HT. Não houve diferença entre a porcentagem de fibrose nos animais dos grupos HT e CS, demonstrando um efeito benéfico do treinamento resistido na prevenção da remodelação cardíaca patológica.

### Função contrátil de miócito simples

Sob estimulação elétrica, miócitos dos animais do grupo HS apresentaram uma relação contração-frequência positiva nas frequências 1, 3 e 5 Hz; a uma grandeza menor de encurtamento do que os animais dos grupos CS e HT ( Tabela 2 ). Essa diferença perdeu seu valor estatístico de 5 para 7 Hz, onde a relação contração-frequência se tornou negativa. Além disso, a velocidade de partida (um índice de velocidade de contração) foi mais lenta nas células do grupo HS do que no grupo do CS acima da faixa de 1 a 7 Hz. Entretanto, na comparação com o grupo HT, a velocidade mais baixa foi encontrada apenas em 1, 3 e 7 Hz. Da mesma forma, a velocidade de retorno (um índice da velocidade de relaxamento) foi mais baixa no grupo HS do que no grupo de CS. Na comparação com o grupo HT, a velocidade mais baixa foi encontrada apenas em 1 e 3 Hz.

**Tabela 2:** Efeito do treinamento físico resistido na contração e relaxamento do miócito do ventrículo esquerdo

	CS	HS	HT
	Mediana (FIQ 25%-75%)	Mediana (FIQ 25%-75%)	Mediana (FIQ 25%-75%)
**Encurtamento (% c.c.r.)**			
FE (1 Hz)	7,69 (5,74-9,42)	5,26 (3,23-7,08)*	7,89 (5,80-9,30) ^†^
FE (3 Hz)	8,02 (5,47-10,14)	6,08 (3,73-8,29)*	7,70 (6,59-10,11) ^†^
FE (5 Hz)	8,16 (6,06-10,15)	6,74 (4,83-8,78)*	8,26 (6,25-10,20) ^†^
FE (7 Hz)	7,32 (4,86-9,33)	6,04 (4,37-8,01)	6,95 (5,41-8,90)
**Velocidade de partida**			
FE (1 Hz)	262,9 (191,8-330,3)	189,2 (106,1-266,8)*	250,7 (179,1-307,0) ^†^
FE (3 Hz)	317,7 (222,6-411,2)	250,2 (129,3-332,6)*	288,5 (226,4-416,1) ^†^
FE (5 Hz)	365,8 (246,7-473,2)	303,3 (175,5-417)*	342,4 (254,7-467,3)
FE (7 Hz)	369,8 (284,4-472,9)	322,2 (209,7-367,9)*	344,9 (293,1-469,5) ^†^
**Velocidade de retorno**			
FE (1 Hz)	229,0 (158,2-282,5)	143,6 (76,53-220,7)*	206,5 (148,4-274,1) ^†^
FE (3 Hz)	254,6 (177,2-321,3)	191,9 (97,98-254,2)*	241,3 (159,2-323,8) ^†^
FE (5 Hz)	273,3 (218,4-354,5)	236,1 (126,9-279,6)*	247,6 (178,4-353,6)
FE (7 Hz)	285,2 (226,9-362,6)	234,3 (153,5-293,8)*	260,5 (202,9-356,9)

Os valores são apresentados como medianas acompanhadas da faixa interquartil (FIQ) de 10 células por animal em cada grupo (n=7 ratos em cada grupo). % c.c.r., porcentagem do comprimento da célula em repouso; FE: frequência de estimulação; CS: controle sedentários; HS: hipertensão com sedentarismo; HT: hipertensão com treinamento. ^*^ p <0,05 vs. CS; ^**^ p <0,01 vs. CS; ^†^ p <0,05 vs. HS. Kruskal-Wallis, seguido de teste post-hoc de Dunn.

## Discussão

O presente estudo examinou se o TFR de intensidade baixa a moderada poderia demonstrar ser benéfico para as funções contráteis de VE e miócitos em ratos durante o desenvolvimento de HAP induzida por MCT. Nossos achados demonstram pela primeira vez que ratos tratados com MCT (duas injeções de MCT de 20 mg/kg, com intervalo de 7 dias) subiram escada durante o desenvolvimento da HAP e aumentaram progressivamente sua tolerância ao esforço físico. O índice de tolerância ao esforço físico do nosso estudo, a máxima carga carregada, foi progressivamente mais alto no grupo HT em comparação com os grupos HS e CS durante o experimento. O modelo de TFR foi eficiente no aumento da força muscular e outro modelo de hipertensão de ratos. ^44^ O aumento no peso corporal e na máxima carga carregada observado aqui sugere um efeito protetor do TFR contra a perda e disfunção e perda de músculo esquelético. Esse é um achado interessante desde que a sarcopenia, a intolerância ao esforço físico, e a letargia foram relatadas como características desse modelo de HAP. ^42 , 45 - 47^ A potência muscular demonstrou melhorar em pacientes com HAP em resposta a programas de exercícios combinados (aeróbicos + resistidos). ^23 - 25^ Além disso, o aumento da força muscular é importante para indivíduos hipertensos, já que alivia a sobrecarga cardiovascular durante suas atividades rotineiras e foi associado à proteção contra mortalidade global. ^48^

O programa de TFR usado no presente estudo expandiu o tempo até os animais exibirem sinais de possível surgimento de insuficiência cardíaca (ou seja, ponto de desfecho). Embora não haja estudo sobre os efeitos do modelo de TFR em tal ponto de desfecho em ratos com HAP induzida por MCT, o prolongamento do ponto de desfecho em ratos injetados com MCT em resposta à corrida voluntária foi relatado por nosso grupo, ^17 , 18^ e a sobrevida estendida em resposta a corrida na esteira foi demonstrada por outros, ^45 , 49^ mais especificamente quando iniciada nos estágios iniciais da doença. A sobrevida melhorada também foi demonstrada em pacientes com HAP submetidos a intervenções de exercícios combinados (aeróbicos + resistidos). ^23 - 25^

Nosso regime de TFR beneficiou os parâmetros funcionais e estruturais do VE em ratos injetados com MCT. Em relação à função do ventrículo esquerdo, a ecocardiografia demonstrou que 42,86% dos ratos sedentários injetados com MCT (grupo HS) tiveram fração de ejeção abaixo de 50%, e 28,57% apresentaram encurtamento fracional abaixo de 25%, o que indica disfunção do ventrículo esquerdo. Entretanto, os animais treinados (grupo de HT) a presença da disfunção do ventrículo esquerdo foi mais baixa nos ratos sedentários (grupo HS), o que sugere uma função protetora do exercício de resistência. Esses achados estão alinhados com as alterações causadas pelo TFR empregado no tecido do VE. Por exemplo, o TFR aumentou a porcentagem de colágeno tipo III ao mesmo tempo em que reduziu a porcentagem de colágeno tipo I e fibrose nos ratos com HAP induzida por MCT, demonstrando, portanto, o efeito protetor desse regime de exercícios contra a disfunção do ventrículo esquerdo e a remodelação adversa levando à atenuação da progressão da HAP.

Os parâmetros de órgãos demonstraram que ratos sedentários injetados com MCT (grupo HS) apresentou valores do ventrículo direito (ou seja, peso do ventrículo direito, índice de Fulton e proporção entre peso do ventrículo direito e comprimento da tíbia) e pulmão (ou seja, peso do pulmão, proporção entre peso do pulmão e comprimento da tíbia) mais altos do que o grupo de CS. Apesar de não haver nenhuma mudança no peso do VE e na proporção entre VE e comprimento da tíbia, o comprimento, a largura e o volume de miócitos simples diminuíram pela MCT (CS > HS). Entretanto, o TFR evitou esse tipo de redução nas dimensões da célula (HT = CS), o que indica a manutenção da massa do ventrículo esquerdo e sugere o efeito protetor do programa de TFR aplicado contra a remodelação adversa do ventrículo esquerdo.

Juntamente com a redução das dimensões dos miócitos, a MCT induziu a disfunção contrátil de miócitos simples. Miócitos do grupo HS tiveram encurtamento menor e velocidades de contração e relaxamento mais altas do que o grupo CS. Mais importante, o TFR atenuou a disfunção contrátil, já que esses parâmetros celulares do grupo HT foram semelhantes aos do grupo CS, indicando, assim, melhorias na função contrátil dos miócitos do grupo HT em relação aos do grupo HS. As proteínas reguladoras do cálcio (ou seja, receptor de rianodina 2; fosfolambano e a ATPase 2 do retículo sarcoplasmático gerenciam a força e o tempo da contração de cardiomiócitos e são relatadas como reguladas no ventrículo direito de ratos tratados com MCT. ^15 , 45^ Ainda são necessárias investigações posteriores sobre se o regime de TFR empregado aumenta a expressão e a atividade dessas proteínas, embora esse efeito de exercício tenha sido demonstrado em ratos saudáveis normotensos. ^50 , 51^

Considerados juntos, nossos resultados demonstram que o TFR empregado durante o desenvolvimento da HAP induzida por MCT foi benéfico à estrutura e à função do ventrículo esquerdo e dos miócitos, o que resultou em melhoria da tolerância ao esforço físico e do tempo até o possível surgimento de insuficiência cardíaca nos animais.

A partir das recomendações de treinamento resistido para pacientes com doenças cardiovasculares, ^20^ utilizamos a intensidade de baixa a moderada. Considerando que se relata que o exercício de alta intensidade promove os maiores benefícios em pacientes com insuficiência cardíaca ^52^ e que os ratos injetados com MCT treinaram com carga progressiva até o tempo mediano do ponto de desfecho dos ratos sedentários (28 dias), pode ser possível aumentar experimentalmente a intensidade do treinamento usando-se técnicas de recompensa para aumentar os efeitos do treinamento resistido.

Por fim, este estudo tem limitações. Primeiramente, a velocidade da subida não é controlada neste modelo. Segundo, a duração do período de treinamento está limitada pelos efeitos da MCT. Apesar disso, nossos resultados demonstraram efeitos positivos do programa de treinamento resistido no tempo de possível surgimento de insuficiência cardíaca, tolerância ao esforço físico e disfunção do VE.

## Conclusão

Nossos achados demonstram que junto com o aumento do tempo para possível surgimento de insuficiência cardíaca e na tolerância ao esforço físico, o treinamento resistido de intensidade baixa a moderada atenua o desenvolvimento de disfunções do ventrículo esquerdo no modelo da HAP induzida por MCT. Portanto, o TFR de intensidade baixa a moderada é benéfico para funções contráteis do ventrículo esquerdo e dos miócitos nesse modelo. Esses resultados têm relevância clínica, já que corroboram os benefícios de saúde do treinamento resistido em indivíduos com doença cardiopulmonar, incluindo HAP. Sugerimos que o treinamento resistido de intensidade baixa a moderada seja testado em pacientes com HAP.
