# Supplementary material for: O Treinamento Físico Resistido Atenua as Disfunções Ventriculares Esquerdas em Modelo de Hipertensão Arterial Pulmonar
Source: Arq Bras Cardiol. 2022 Aug 24;119(4):574–84. [Article in Portuguese] doi: 10.36660/abc.20210681 (PMC9563884; doi:10.36660/abc.20210681)
Supplement: Translation

# Translation: Resistance Exercise Training Mitigates Left Ventricular Dysfunctions in Pulmonary Artery Hypertension Model

**Authors:** Leôncio Lopes Soares, Luciano Bernardes Leite, Luiz Otávio Guimarães Ervilha, Bruna Aparecida Fonseca da Silva, Maíra Oliveira de Freitas, Alexandre Martins Oliveira Portes, Leonardo Mateus Teixeira Rezende, Filipe Rios Drummond, Miguel Araújo Carneiro, Mariana Machado Neves, Emily Correna Carlo Reis, Antônio José Natali

**Affiliations:** 1 Brasil Universidade Federal de Viçosa, Departamento de Educação Física, Viçosa, MG – Brasil; 2 Brasil Universidade Federal de Viçosa, Departamento de Biologia Geral, Viçosa, MG – Brasil; 3 Brasil Universidade Federal de Viçosa, Departamento de Medicina Veterinária, Viçosa, MG – Brasil

**Keywords:** Heart Failure, Pulmonary Hypertension, Rats, Physical Conditioning, Animal/methods, Myocytes, Cardiac, Ventricular Dysfunction, Left, Exercise

## Abstract

**Background:**

The right ventricular hypertrophy and dilation observed in pulmonary artery hypertension (PAH) damages the left ventricle (LV) dynamics by flattening the interventricular septum.

**Objective:**

To investigate whether low- to moderate-intensity resistance exercise training (RT) is beneficial to LV and cardiomyocyte contractile functions in rats during the development of monocrotaline (MCT)-induced PAH.

**Methods:**

Male Wistar rats (Body weight: ~ 200 g) were used. To assess the time to potential heart failure onset (i.e., end point), rats were divided into sedentary hypertension until failure (SHF, n=6) and exercise hypertension until failure (EHF, n=6) groups. To test RT effects, rats were divided into sedentary control (SC, n = 7), sedentary hypertension (SH, n=7), and exercise hypertension (EH, n=7) groups. PAH was induced by two MCT injections (20 mg/kg, with 7 days interval). Exercise groups were submitted to an RT protocol (Ladder climbing; 55-65% of carrying maximal load), 5 times/week. Statistical significance was assumed at P < 0.05.

**Results:**

RT prolonged the end point (~25 %), enhanced the physical effort tolerance (~ 55%), and mitigated the LV and cardiomyocyte contractility dysfunctions promoted by MCT by preserving the ejection fraction and fractional shortening, the amplitude of shortening, and the velocities of contraction and relaxation in cardiomyocytes. RT also prevented increases in left ventricle fibrosis and type I collagen caused by MCT, and maintained the type III collagen and myocyte dimensions reduced by MCT.

**Conclusion:**

Low- to moderate-intensity RT benefits LV and cardiomyocyte contractile functions in rats during the development of MCT-induced PAH.

## Introduction

Increases in the pulmonary vasculature resistance, mainly caused by endothelial dysfunction, leads to pulmonary arterial hypertension (PAH).^1^ The chronic pulmonary vasculature resistance overloads the right ventricle (RV), resulting in pathological remodeling,^2^ and dysfunction because of hypertrophy and dilation.^1^ Such remodeling affects the left ventricle (LV) dynamics because of the direct ventricular interaction. In this framework, the left ventricle dynamics are damaged by the interventricular septum flattening,^3 , 4^ as it faces impaired early diastolic filling, reduced end-diastolic volume, and adverse remodeling.^3 , 5 , 6^ Therefore, PAH patients exhibit reduced stroke volume^3^ and physical effort tolerance, which negatively impacts their quality of life and survival.^7^

Pharmacological therapies aim to reduce pulmonary artery pressure and the overload to the RV, thereby maintaining the cardiac function.^8^ It has been demonstrated that patients with PAH may maintain the cardiac function by non-pharmacological means, such as practicing regular physical exercise.^9 , 10^ In the experimental model of monocrotaline (MCT)-induced severe PAH, for example, previous and early aerobic exercise have been shown to promote cardiovascular benefits, such as mitigation of right ventricular hypertrophy, dysfunction, and adverse remodeling.^11 - 16^ Our research group^17 , 18^ recently reported that voluntary running (i.e. intermittent high-intensity exercise) postpones the onset of heart failure, and lightens RV e adverse remodeling and myocyte dysfunction (i.e. myocyte contractility and intracellular Ca^2^cycling deterioration) in this model. Furthermore, our study also demonstrated that moderate-intensity continuous aerobic exercise prevents right ventricle adverse remodeling and myocyte contractility and Ca^2^cycling impairments.^19^

The use of low- to moderate-intensity resistance exercise training (RT) has been recommended to compose exercise programs to promote health and prevent cardiovascular diseases,^20 , 21^ including those related to left ventricular dysfunction.^22^ Regarding PAH, combined exercise interventions, including aerobic, resistance, and specific inspiratory muscle training proved safe for these patients and yielded significant improvements in muscle power, exercise capacity, and survival.^23 - 25^ Nevertheless, while aerobic exercise has been demonstrated to prevent left ventricular systolic and diastolic dysfunction in both baseline and isovolumic conditions^26^ in MCT-induced PAH, the impact of RT in the left ventricular dysfunction in this model is unknown.

While the animal models have supported the discovery of new therapies and the understanding of PAH pathophysiology, the model of MCT lung injury in rodents using the injection of 60 mg/kg body mass induces severe PAH in a subacute process, which is limited in order to simulate human chronic PAH.^27^ In this sense, Whang et al.^28^ demonstrated that 40 mg/kg MCT divided into two injections of 20 mg/kg with an interval of seven days better mimics chronic PAH with those common changes in the structure and function of pulmonary arteries and RV observed in humans. Therefore, the present study applied this model in rats to test whether low- to moderate-intensity RT might prove beneficial to LV and myocyte contractile functions during the development of MCT-induced PAH. The hypothesis of this study is that low- to moderate-intensity RT is beneficial to LV and myocyte contractile functions in rats during the development of MCT-induced PAH.

## Methods

### Experimental design and PAH induction

After the definition of the sample size,^29^ thirty-three male Wistar rats [Body weight: ~200 g] were obtained from the animal laboratory at the Federal University of Viçosa, MG, Brazil. The animals were housed in transparent polycarbonate cages, kept in a room with a controlled temperature (~22 °C) and ~60% relative humidity, under a 12/12 h light/dark cycles, and had free access to water and commercial chow.

To assess the time to the onset of potential heart failure, 12 animals (~200 g) were divided into two groups, by using simple randomization: sedentary hypertension until failure (SHF, n = 6) and exercise hypertension until failure (EHF, n = 6). After the MCT injections, rats from the SHF and EHF groups were euthanized when they showed previously validated external clinical signs of potential heart failure onset (e.g., weight loss, dyspnea, piloerection) and could no longer feed properly, climb the ladder (EHF group), or even move in the cage,^30 - 37^ which was considered the end point.

To test whether RT is beneficial during the development of PAH, 21 animals (~200 g) were divided into groups using blocked randomization: sedentary control (SC, n = 7), sedentary hypertension (SH, n = 7), and exercise hypertension (EH, n = 7). Animals from the SH, EH, and SC groups were euthanized at the median end point day (± 1 day) of the SHF animals (i.e., 28 days). The median time to the onset of potential heart failure represented the moment after MCT treatment when more than 50% of the group reached the end point day. The animals in the exercise groups were submitted to RT while those in sedentary groups were maintained in their cages.

To induce PAH, animals from the SHF, EHF, SH, and EH groups received 2 intraperitonial MCT (Sigma-Aldrich, USA) injections of 20 mg/kg, at a 7-day interval, to induce right ventricular failure.^28^ Control animals received equivalent volume injections of saline.

Experiments were conducted in accordance with international procedures for animal research (Scientific Procedures; Act 1986). All protocols were reviewed and approved by the Institutional Ethics Committee (protocol number 02/2019).

### Resistance training and maximal load test

The animals were familiarized to the RT protocol (adapted from Hornberger and Farrar^38^ ) for one week before the first MCT or saline injection, with no additional load. RT consisted of climbing a ladder (1.1 m high; 80º inclination) with 2 min resting intervals, with the load based on a maximal carrying load test. The maximal carrying load test was performed before MCT or saline injection (time 0) and on the 14^th^, 21^st^, and 28^th^day after injections. The test consisted of ladder climbing with an initial load of 75% of body weight, which was progressively increased by an additional 15% in the subsequent climbs until the animal could no longer climb.^39^ The load was fixed on the rat’s tail, and climbs were interspersed with 2 min resting intervals. The maximal carrying load was used as the physical effort tolerance index.

Exercised animals were submitted to a RT program, 5 times/week during the experimental period until the day before euthanasia, totaling twenty exercise sessions. RT load was 55-65% of the maximal carrying load, following the recommendations for patients with cardiovascular diseases.^20^ Each training session consisted of 15 climbs, interspersed with 60-second intervals, with the training load adjusted after the maximal carrying load tests (14^th^and 21^st^day).

### Echocardiography and sample collection

The echocardiographic evaluations were performed on the 28^th^day after the first MCT injection. The animals were anesthetized (Isoflurane 1.5% and 100% oxygen in a constant flow of 1L/min; Isoflurane, BioChimico, Brazil), and the images were obtained while the animals remained in the lateral decubitus position. Two-dimensional studies with a fast-sampling rate of 120 fps in M-mode were performed using the MyLabTM30 ultrasound system (Esaote, Genoa, Italia) and 11 MHz nominal frequency transducers. The two-dimensional transthoracic echocardiography and M-mode was obtained at a scanning speed of 200 mm adjusted according to the heart rate.^40^ To evaluate LV function, the following parameters were assessed: LV ejection fraction (EF) and fractional shortening (FS). To characterize the PAH, the tricuspid annular plane systolic excursion (TAPSE) was determined.

At the median end point day (± 1 day) of the SHF animals, animals from SH, EH, and SC groups were euthanized. After euthanasia, animals from SC, SH, and EH groups had the heart, ventricles, and lungs dissected, weighed, and processed for analyses of interest, as described below. The right tibia was dissected, and its length was measured.

### Histomorphometry

The histological analyses of the LV were performed as previously described.^41 , 42^ Briefly, immediately after collection, fragments of the LV were fixed on the Karnovsky fixator (paraformaldehyde 4% and glutaraldehyde 4% in 0.1M phosphate buffer, pH 7.4) for 24 hours. The fragments were then dehydrated in ethanol, clarified in xylol, and embedded in paraffin. Blocks were cut into 5 μm-thick sections, mounted on histological slides, and stained with Hematoxylin & Eosin to measure the cross-sectional area (CSA), or with Sirius Red to count collagen fibers and/or with Masson’s trichrome for cardiac fibrosis count. To avoid repeated analyses of the same histological area, the sections were evaluated in semi-series, using one in every 10 sections. Digital images from Sirius Red stained slides were obtained using a polarized light microscope (Olympus AX-70, Tokyo, Japan) connected to a digital camera (Olympus Q Color-3, Tokyo, Japan), and images of slides stained with Hematoxylin & Eosin and Masson’s trichrome were obtained using a light microscope (Olympus AX-70, Tokyo, Japan) connected to a digital camera (Olympus Q Color-3, Tokyo, Japan). The quantification of collagen types and cardiac fibrosis was performed using a specific color identification tool using the Image-pro Plus 4.5 software (Media Cybernetics, Silver Spring, MD, USA). Myocyte CSA was measured using a specific tool (manual measurement in software image pro-plus 4.5).

### Isolation of left ventricle myocytes

The heart was attached to a Langendorff-retrograde perfusion system, and single LV myocytes were isolated as previously described.^18^ Briefly, the heart perfused system via aorta with Tyrode solution containing (in mM; Sigma-Aldrich, USA): 130 NaCl, 1.43 MgCl2, 5.4 KCl, 0.75 CaCl2, 5.0 Hepes, 10.0 glucose, 20.0 taurine, and 10.0 creatine, pH 7.4 until for about 5 min. The Tyrode solution was exchanged to Tyrode solution containing EGTA (0.1 mM) for 6 min. The heart was then perfused with Tyrode solution containing 1 mg/ml collagenase type II (Worthington, USA) and 0.1 mg/ml protease (Sigma-Aldrich, USA) for about 12 min. Next, the LV of the digested heart was removed and cut into small fragments, which were placed into a conical flask containing the enzymatic solution (collagenase and protease). The cells were mechanically separated by shaking the flask for 5 min. The dispersed cells were separated from the non-dispersed tissue by filtration through centrifugation. The isolated cells were stored at 5°C until use. Isolated myocytes were used within 2 to 3 hours after isolation. The solutions used in the isolation procedure were oxygenated (O_2_100% - White Martins, Brazil) and maintained at 37ºC.

### Single myocyte contractile function

The contractile function of LV myocytes was measured using an edge detection system (Ionoptix, Milton, USA) mounted on an inverted microscope (Nikon Eclipse - TS100, Japan) as previously described.^19^ Myocytes were placed in a bath on the stage of an inverted microscope and superfused with Tyrode’s solution containing, in mM (Sigma-Aldrich, USA): 137 NaCl, 5.4 KCl, 0.33 NaH_2_PO_4_, 0.5 MgCl_2_, 5 HEPES, 5.6 glucose 1.8 CaCl_2,_pH 7.4 with 5N NaOH, at 37˚C. Only myocytes exhibiting a clear, regular striation (sarcomere) pattern, with no spontaneous contraction in the absence of external stimulation, and responding to 1Hz stimulation with a single twitch were tested. Myocytes were stimulated (Myopacer, Ionoptix, Milton, USA) to contract at a progressive stimulation frequency (1, 3, 5 and 7 Hz) using external electrodes, and the resultant cell shortening was measured by analyzing a video image of the cell using Ionoptix camera and software (Ionoptix, Milton, MA, USA). Cell shortening was expressed as % of resting cell length.

The myocyte length and width were obtained from the video image of the cell; and the cell volume was calculated as previously described.^43^

### Statistical analysis

The normality of the data was tested using the Shapiro-Wilk test. Data are presented as mean ± SD for continuous variables with normal distribution and median accompanied by the interquartile range for continuous variables without normal distribution. The end point, ventricular remodeling parameters, and contractile parameters of isolated cardiomyocytes showed a non-normal distribution, while exercise parameters, body and organ weight, and cross-sectional area and isolated cell morphometry presented normal distribution. The end point was tested by the Kaplan-Meier curve analysis through the Log-rank test. Maximum carrying load, body weight, LV function, organ weight, and single cell parameters were tested by one-way analysis of variance (ANOVA) or Kruskal-Wallis followed by the Dunn’s post hoc test. Maximum carrying load was tested by one-way repeated measures ANOVA. ANOVAs were followed by the pairwise Tukey correction test. Person’s Chi-squared test (X^2^) was used to assess the proportion of animals that presented intraventricular septum flattening. Statistical significance was assumed at P < 0.05. Data description, numbers of rats and myocytes are given in the table and figure legends. All analyses were performed using GraphPad Prism, version 6.01 (San Diego, CA, USA).

## Results

### Onset of potential heart failure and physical effort tolerance

Figure 1A illustrates that animals from the EHF group performed the resistance exercise protocol during the development of PAH until presenting signs of the onset of potential heart failure. The maximal carrying load increased progressively until day 21, and afterwards it decreased to the initial level on the 35^th^day after the first MCT injection. All animals from both SHF and EHF groups presented signs of the onset of potential heart failure - end point ( Figure 1B ); however, animals in the EHF group had a longer median end point time (37 days) than did those in SHF group (28 days), indicating benefits of resistance exercise.

**Figure 1:** Effect of resistance training on the onset of potential heart failure (end point) and physical effort tolerance. (A) Relative maximal carrying load of hypertensive animals until failure, determined by the maximal carrying load normalized to body weight, at pre-injection (day 0) and on the 14th, 21st, 28th, and 35th day after the first monocrotaline injection. (B) End point, measured in days to present signs of the onset of potential heart failure, was significantly shorter in sedentary hypertension until failure (SHF, n = 6) than in exercise hypertension until failure (EHF, n = 6) rats. **P < 0.01, Kaplan-Meier curve analysis by the Log-rank test. (C) Relative maximal carrying load of control, hypertensive sedentary and exercise animals, determined as in panel A. Exercise hypertension (EH, n=7) rats exhibited higher carrying load gain than sedentary control (SC, n=7) and sedentary hypertension (SH, n=7) from the 14th day on. Repeated measures ANOVA followed by Tukey correction test. aP < 0.05 vs. SH; bP < 0.05, vs. SC; *P < 0.05 vs. Before MCT injection.

Hypertensive rats from the EH group improved their tolerance to physical effort ( Figure 1C ) throughout the experiment. The maximum carrying load in the EH group was higher on days 21 and 28 than on day 0. Moreover, these animals had a higher maximum carrying load on the 14^th^, 21^st^and 28^th^day, when compared to those in the SC and SH groups.

### Left ventricular function and morphology

The echocardiographic evaluation showed a flattening of the interventricular septum, called the D-shaped left ventricle, in animals from the SH and EH groups ( Figure 2A ), which suggests RV pressure overload, characteristic in PAH. Such a morphological change was greater in the SH than in the SC group, while EH presented intermediate values ( Figure 2D ). Regarding the left ventricle function, no difference between the groups for ejection fraction ( Figure2B ) and fractional shortening ( Figure 2C ) was found. Despite that, it is important to note that 3 out of 7 animals in the SH group presented an ejection fraction < 50%, and 3 out of 7 presented fractional shortening < 25%, indicative of left ventricular failure. By contrast, none of the exercised animals (EH), in the same period, showed an ejection fraction < 50%, and only 1 out of 7 presented fractional shortening < 25%.

**Figure 2:** Effect of resistance exercise training on left ventricular function assessed on the 28th day after the first monocrotaline injection. (A) Representative echocardiograph images. (B) Ejection fraction. (C) Fractional shortening. (D) D-shaped left ventricle. Values are means ± SD (n = 7 rats in each group). SC: sedentary control; SH: sedentary hypertension; EH: exercise hypertension; RV: right ventricle; LV: left ventricle. Dotted line indicates limits for the classification of impaired function. Panel B and C: One-Way ANOVA followed by the Tukey’s post hoc test. Panel D: Pearson’ s Chi-squared test (x2 test). *p < 0.05.

The presence of PAH in animals from the SH group was also characterized by the TAPSE values. Animals from the SH group exhibited lower TAPSE values (1.43 ± 0.23) than did those from the SC (2.06 ± 0.17) and EH (2.13 ± 0.36) groups.

Animals from the SH group presented lower body weight than did those from the SC and EH groups ( Table 1 ). Despite no difference between group for heart weight, animals from the SH and EH groups had higher RV weight and right ventricle-to-tibia ratio than did animals in the SC group, which indicates RV hypertrophy. While lung weight and lung weight-to-tibia ratio were higher in the SH and EH groups than in the SC group, no difference between groups for LV weight and LV weight-to-tibia ratio was found. Regarding left ventricle myocyte dimensions, the SH group presented a lower length, width, and volume than did the SC group. The EH group presented intermediate values between the SC and SH groups. Animals of the SH groups exhibited lower CSA when compared to animals in the SC and EH groups. By contrast, there was no difference in the CSA of animals in the EH group when compared to animals in the SC group, suggesting a beneficial effect of the resistance exercise program in preventing adverse left ventricle remodeling.

**Table 1:** Effect of resistance exercise training on body and organ weights

	SC	SH	EH
Final BW (g)	298.6 ± 19.01	276.3 ± 19.87^*^	303.7 ± 20.98^†^
Heart weight (g)	1.23 ± 0.11	1.30 ± 0.18	1.28 ± 0.12
RV weight (g)	0.33 ± 0.04	0.42 ± 0.03^*^	0.44 ± 0.05^*^
LV weight (g)	0.74 ± 0.10	0.65 ± 0.07	0.70 ± 0.07
Lung weight (g)	1.65 ± 0.28	2.77 ± 0.41**	2.38 ± 0.33**
Ratio of RV weight to tibia length (g/cm)	0.09 ± 0.01	0.11 ± 0.00*	0.11 ± 0.01*
Ratio of LV weight to tibia length (g/cm)	0.20 ± 0.02	0.17 ± 0.02	0.18 ± 0.02
Ratio of lung weight to tibia length (g/cm)	0.45 ± 0.10	0.73 ± 0.11**	0.63 ± 0.09*
Myocyte length (µm)	132.3 ± 19.09	122.5 ± 19.86**	129.2 ± 21.42
Myocyte width (µm)	46.12 ± 10.08	41.75 ± 9.95*	43.64 ± 9.50
Myocyte volume (pL)	46.24 ± 3.97	38.71 ± 3.18**	42.62 ± 3.61
Myocyte CSA (µm^2^)	462.1 ± 21.86	400.5 ± 43.34*	492.2 ± 66.56^†^

Data are mean ± SD of 7 rats and 10 cells in each group; SC: sedentary control; SH: sedentary hypertension; EH: exercise hypertension; BW: body weight; RV: right ventricle; LV: left ventricle; *p < 0.05 vs. SC; **p < 0.01 vs. SC; †p < 0.05 vs. SH. One-way ANOVA followed by the Tukey post hoc test.

### Left ventricular adverse remodeling

Figure 3 shows data on LV collagen fibers and fibrosis. Hypertensive animals (SH and EH) presented a higher percentage of type I collagen compared to animals in the control group (SC) ( Figure 3A ). However, animals in the EH group had a lower percentage of type I collagen when compared to animals in the SH group, showing the protective effect of RT on the progression of PAH. In addition, animals in the EH group exhibited a higher percentage of type III collagen than those in the sedentary animals (SC and SH) ( Figure 3B ). Hypertensive animals (SH and EH) had a higher percentage of total collagen, compared to animals in the control group ( Figure 3C ). Concerning LV fibrosis ( Figure 3D ), animals in the SH group presented a higher percentage, compared to the those in the SC and EH groups. There was no difference between the percentage of fibrosis in animals from the EH and SC groups, showing a beneficial effect of resistance exercise on the prevention of pathological cardiac remodeling.

**Figure 3:** Effect of resistance exercise training on left ventricle remodeling. (A) Percentage of type I collagen. (B) Percentage of type III collagen. (C) Percentage of total collagen. (D) Percentage of fibrosis in the LV. (E) Representative photomicrographs of LV tissue stained with Sirius Red; (F) Representative photomicrographs of LV tissue stained with Masson’s trichrome. Blue arrow indicates type I collagen; White arrow indicates type III collagen; Black arrow indicates cardiac fibrosis. Values are presented as median accompanied by the interquartile range of 10 images per animal in each group (n = 5 rats in each group). SC: sedentary control; SH: sedentary hypertension; EH: exercise hypertension. Kruskal-Wallis, followed by the Dunn’s post hoc test: * P < 0.05, and ** p < 0.01.

### Single myocyte contractile function

Under electrical stimulation, myocytes from SH animals showed a positive contraction-frequency relationship at the frequencies of 1, 3, and 5 Hz and a lower magnitude of shortening than those from the SC and EH groups ( Table 2 ). Such a difference lost its statistical difference from 5 to 7Hz, where the contraction-frequency relationship became negative. In addition, the departure velocity (an index of contraction velocity) was slower in cells from the SH group than in those from the SC group over the range of 1-7 Hz. However, when compared to the EH, the lower speed was found only at 1, 3, and 7 Hz. Likewise, the return velocity (an index of relaxation velocity) was slower in the SH group than in the SC group. When compared to the EH, the slower speed was found only at 1 and 3 Hz.

**Table 2:** Effect of resistance exercise training on left ventricular myocyte contraction and relaxation

	SC	SH	EH
	Median (IQR 25%-75%)	Median (IQR 25%-75%)	Median (IQR 25%-75%)
**Shortening (% r.c.l.)**			
S.F. (1 Hz)	7.69 (5.74-9.42)	5.26 (3.23-7.08)*	7.89 (5.80-9.30)^†^
S.F. (3 Hz)	8.02 (5.47-10.14)	6.08 (3.73-8.29)*	7.70 (6.59-10.11)^†^
S.F. (5 Hz)	8.16 (6.06-10.15)	6.74 (4.83-8.78)*	8.26 (6.25-10.20)^†^
S.F. (7 Hz)	7.32 (4.86-9.33)	6.04 (4.37-8.01)	6.95 (5.41-8.90)
**Departure velocity**			
S.F. (1 Hz)	262.9 (191.8-330.3)	189.2 (106.1-266.8)*	250.7 (179.1-307.0)^†^
S.F. (3 Hz)	317.7 (222.6-411.2)	250.2 (129.3-332.6)*	288.5 (226.4-416.1)^†^
S.F. (5 Hz)	365.8 (246.7-473.2)	303.3 (175.5-417)*	342.4 (254.7-467.3)
S.F. (7 Hz)	369.8 (284.4-472.9)	322.2 (209.7-367.9)*	344.9 (293.1-469.5)^†^
**Return velocity**			
S.F. (1 Hz)	229.0 (158.2-282.5)	143.6 (76.53-220.7)*	206.5 (148.4-274.1)^†^
S.F. (3 Hz)	254.6 (177.2-321.3)	191.9 (97.98-254.2)*	241.3 (159.2-323.8)^†^
S.F. (5 Hz)	273.3 (218.4-354.5)	236.1 (126.9-279.6)*	247.6 (178.4-353.6)
S.F. (7 Hz)	285.2 (226.9-362.6)	234.3 (153.5-293.8)*	260.5 (202.9-356.9)

Data are presented as median accompanied by the interquartile range (IQR) of 10 cells per animal in each group (n = 7 rats in each group). % r.c.l., percentage of resting cell length; SF: stimulation frequency; SC: sedentary control; SH: sedentary hypertension; EH: exercise hypertension. *p < 0.05 vs. SC; **p < 0.01 vs. SC; †p < 0.05 vs. SH. Kruskal-Wallis, followed by the Dunn’s post hoc test.

## Discussion

The present study examined whether low- to moderate-intensity RT might prove beneficial to LV and myocyte contractile functions in rats during the development of MCT-induced PAH. Our findings demonstrate for the first time that rats treated with MCT (Two MCT injections of 20 mg/kg, at a 7-day interval) climbed the ladder during the development of PAH and progressively increased their tolerance to physical effort. Our study’s index of physical effort tolerance, the maximal carrying load, was progressively higher in the EH group compared to the SH and SC groups throughout the experiment. This model of RT was efficient in increasing muscle strength in another rat model of hypertension.^44^ The increase in body weight and maximal carrying load observed here suggests a protective effect of RT against skeletal muscle loss and dysfunction. This is an interesting finding since sarcopenia, intolerance to physical effort, and lethargy are reported characteristics of this PAH model.^42 , 45 - 47^ Muscle power has proven to be improved in PAH patients in response to combined exercise (Aerobic + Resistance) programs.^23 - 25^ Moreover, the increase in muscle strength is important for hypertensive individuals, as it lightens the cardiovascular overload during their daily life activities and has been associated with protection against all-cause mortality.^48^

The RT program used in the present study expanded the time until the animals exhibited the signs of the onset of potential heart failure (i.e., end point). Although there is no study on the effects of the RT model on such an end point in rats with MCT-induced PAH, prolonged end point in rats injected with MCT in response to voluntary running has been reported by our group,^17 , 18^ and extended survival in response to treadmill running has been demonstrated by others,^45 , 49^ more markedly when started at the early stages of the disease. Enhanced survival has also been demonstrated in PAH patients submitted to combined exercise (Aerobic + Resistance) interventions.^23 - 25^

Our RT regime benefited the LV functional and structural parameters in MCT-injected rats. Regarding left ventricular function, echocardiography showed that 42.86% of sedentary rats injected with MCT (SH group) had an ejection fraction below 50%, and 28.57% presented fractional shortening below 25%, which indicates left ventricular dysfunction. Nevertheless, in exercised animals (EH group) the presence of left ventricular disfunction was lower than in sedentary rats (SH group), thus suggesting a protective role of resistance exercise. These findings run in line with changes caused by the employed RT in the LV tissue. For instance, RT increased the percentage of type III collagen while it reduced the percentage of type I collagen and fibrosis in rats with MCT-induced PAH, thus showing the protective effect of this exercise regime against left ventricular dysfunction and adverse remodeling leading to mitigation of the PAH progression.

The organ parameters showed that MCT-injected sedentary rats (SH group) exhibited higher RV (i.e. RV weight, Fulton’s index, and RV weight/tibia length ratio) and lung (i.e. Lung weight, lung weight/tibia length ratio) values than did the control group (SC). Despite no change in whole LV weight and LV to tibia length ratio, single myocyte length, width, and volume were decreased by MCT (SC > SH). However, RT prevented this type of cell dimension reduction (EH = SC), which indicates the maintenance of the left ventricular mass and suggests the protective effect of the applied RT program against the left ventricular adverse remodeling.

Along with reduction in myocyte dimensions, MCT induced single myocyte contractile dysfunction. Myocytes from the SH group had lower shortening and longer contraction and relaxation velocities than did those in the SC group. More importantly, RT mitigated the contractile dysfunction as these cell parameters in the EH group were similar to those in the SC group, thus indicating improvements in the contractile function in myocytes from the EH group relative to those from the SH group. The calcium regulatory proteins (i.e., Ryanodine receptor 2, Phospholamban, and Sarcoplasmic reticulum ATPase 2a) manage the force and time course of cardiomyocyte contraction and are reported to be downregulated in the RV of MCT-treated rats.^15 , 45^ Whether the employed RT regime increases the expression and activity of these proteins warrants further investigations, though such an exercise effect has been demonstrated in normotensive healthy rats.^50 , 51^

Taken together, the present study’s results demonstrate that the RT employed during the development of MCT-induced PAH was beneficial to left ventricle and myocyte contractile structure and function, which resulted in enhanced tolerance to physical effort and time to the onset of potential heart failure in the animals.

Considering resistance exercise recommendations for patients with cardiovascular diseases,^20^ the present used low- to moderate-intensity. Considering that high-intensity exercise is reported to promote the highest benefits in patients with heart failure,^52^ and that MCT-injected rats exercised with a progressive load until the median end point time of the sedentary rats (28 days), it might be possible to experimentally increase exercise intensity using reward techniques to expand the resistance exercise effects.

Finally, this study has limitations. First, the speed of climbing is not controlled in this model. Second, the duration of the training period is limited by the effects of MCT. Despite that, our results showed positive effects of the resistance exercise program on both the time to the onset of potential heart failure, physical effort tolerance, and LV dysfunction.

## Conclusion

Our findings demonstrate that along with the increase in the time to the onset of potential heart failure and in the physical effort tolerance, low- to moderate-intensity resistance exercise mitigates the development of left ventricular dysfunctions in the MCT-induced PAH model. Therefore, low- to moderate-intensity RT is beneficial to left ventricular and myocyte contractile functions in this model. These results are of clinical relevance, as they support the health benefits of resistance exercise to individuals with cardiopulmonary disease, including PAH. We suggest that low- to moderate-intensity resistance exercise should be tested in PAH patients.
